# History of Childhood/Adolescence Referral to Speciality Care or Treatment in Adult Patients with Attention-Deficit/Hyperactivity Disorder: Mutual Relations with Clinical Presentation, Psychiatric Comorbidity and Emotional Dysregulation

**DOI:** 10.3390/brainsci13091251

**Published:** 2023-08-27

**Authors:** Giulio Emilio Brancati, Ugo De Rosa, Francesco De Dominicis, Alessandra Petrucci, Alessandro Nannini, Pierpaolo Medda, Elisa Schiavi, Giulio Perugi

**Affiliations:** 1 Department of Clinical and Experimental Medicine, University Hospital of Pisa, 56100 Pisa, Italyalessandro-nannini@virgilio.it (A.N.); 2 Mental Health Centre, Local Health Unit 2, Via San Carlo 2, 06049 Spoleto, Italy; 3 Psychiatry Unit 2, Azienda Ospedaliero-Universitaria Pisana, Via Roma 67, 56126 Pisa, Italy; piermedda@gmail.com (P.M.);

**Keywords:** attention-deficit/hyperactivity disorder, methylphenidate, early treatment, comorbidity, mood disorders, emotional dysregulation

## Abstract

Attention-deficit/hyperactivity disorder (ADHD) is a neurodevelopmental condition that only rarely remits in adulthood. While several studies underlined differences between child and adult ADHD, the relationship between adult clinical presentation and early referral/treatment has been rarely investigated. In our study, 100 adults with ADHD were recruited and subdivided according to a history of referral to speciality care or treatment with methylphenidate (MPH) during childhood/adolescence. The early referral was associated with a history of disruptive behaviors during childhood/adolescence. Current ADHD symptoms were more pronounced in patients first referred during childhood/adolescence but never treated with MPH. Early MPH treatment was associated with lower rates of mood disorders and lower severity of emotional dysregulation at the time of assessment. Negative emotionality mediated the relationship between MPH treatment and mood disorders comorbidity. ADHD patients first referred during childhood/adolescence are characterized by more externalizing features than those first referred in adulthood. MPH treatment during the developmental age may have a role in preventing mood disorders in patients with ADHD, possibly by reducing emotional dysregulation.

## 1. Introduction

Attention-deficit/hyperactivity disorder (ADHD) is a neurodevelopmental disorder, first occurring during childhood, characterized by a peculiar pattern of hyperactivity, inattention, and impulsivity that interferes with functioning or development [1]. ADHD is highly heritable and, in most patients, shows improvement with the administration of dopamine and norepinephrine reuptake inhibitors [1]. Though not included among current diagnostic criteria, symptoms of emotional dysregulation such as affective lability and irritability are often present in ADHD [2,3,4].

For a long time, ADHD has been considered primarily affecting childhood and adolescence. However, a prevalence of about 6%, similar to that observed in children and adolescents, has been estimated in adults [5]. Although the estimation of childhood ADHD persistence is affected by the methods and criteria applied for diagnosis [6], a substantial impact of ADHD symptoms in adulthood has been demonstrated. Early studies showed that partial remission of symptoms after age 18 is observed in about 60% of patients, though most continue to experience impaired functioning [7]. More recently, a longitudinal multi-assessment study found that approximately two-thirds of patients with ADHD fluctuate between remission and recurrence over time, while only less than 10% achieve a sustained remission in adulthood [8]. 

Several studies underlined differences in the clinical picture depending on the age of the patient, showing a reduction of hyperactivity and impulsivity symptoms over time and a predominance of inattentive symptomatology in older populations [7]. Adult ADHD is associated with high unemployment and financial problems, low socioeconomic status, high separation or divorce, and reduced global functioning [9,10]. General mental health symptoms, such as subthreshold anxiety and depression, are also common in adult patients with ADHD, even in the absence of comorbid diagnoses [2]. Patients often exhibit affective symptoms, including low self-esteem, low affect, emotional lability, and irritability, which may be considered a consequence or part of the core ADHD syndrome [4,11,12]. Notably, emotional dysregulation in children with ADHD is associated with a higher persistence of symptoms in adulthood [13,14].

Comorbid psychiatric conditions can also dominate the clinical picture and course of adult ADHD, making it difficult to recognize and properly treat the underlying neurodevelopmental disorder [15,16]. The pattern of ADHD comorbidities changes substantially through the lifespan [17,18]: while in children, oppositional defiant disorder (ODD) and conduct disorder (CD) are the most prevalent comorbid conditions, mood, anxiety and substance use disorders (SUD) become more prevalent during adolescence and adulthood [19,20,21,22]. Even in this case, the presence of psychiatric comorbidities in children with ADHD has been shown to increase the risk for ADHD persistence in adulthood [23]. In turn, ADHD may represent itself as a risk factor for the development of SUD [24], major depression [25,26], and bipolar disorder (BD) [27].

Treatment of ADHD consists of pharmacological, psychoeducational, and psychotherapeutic measures, often combined [11,28]. According to multiple clinical guidelines [29,30], methylphenidate (MPH), an amphetamine derivative, is the first-line pharmacological treatment of ADHD in children/adolescents and adults. Despite the abuse potential of stimulants and ADHD being a risk factor for SUD, there is evidence that MPH treatment may protect against the development of SUD [31,32]. More recent studies also supported the protective role of early ADHD treatment against the development of mood, anxiety, and personality disorders. A retrospective investigation evaluated 75 adolescents with ADHD and found that delayed ADHD pharmacotherapy was associated with a higher risk of subsequent major depressive disorder [33]. In a prospective study on 112 males with ADHD, treatment with stimulants decreased the risk of subsequent depressive, disruptive and anxiety disorders [34]. Lower rates of mood disorders (depression and BD) have also been shown in adults who received stimulant treatment as children compared with untreated individuals [35]. More recently, another large retrospective study showed that ADHD patients treated with stimulants before 18 had a lower prevalence of SUD, anxiety disorders, depressive disorders and personality disorders than those untreated [36]. Finally, a large register-based study from Taiwan’s National Health Insurance database found that ADHD patients who received MPH treatment for at least one year were less likely than untreated patients to be later diagnosed with BD [37].

While differences between treated and untreated patients have been previously evaluated, no studies so far investigated whether clinical differences may also occur between adult ADHD patients who were first referred or diagnosed during the developmental age and those never referred before the age of 18. Indeed, despite the neurodevelopmental nature of the disorder and the importance of early treatment, most adults with ADHD are not referred to psychiatric services during childhood or adolescence and often receive other diagnoses before ADHD is recognized and properly treated [38,39]. The aim of our study was to investigate whether early referral, diagnosis or treatment of ADHD could be related to specific clinical facets and psychiatric comorbidities. We hypothesized that referral to speciality care during childhood/adolescence could be driven by disruptive behaviors, while later diagnosis and treatment may be associated with greater affective and substance use disorders comorbidity. Moreover, to our knowledge, none of the previous studies investigating the effect of early treatment assessed the severity of emotional dysregulation in adults with ADHD. We hypothesize that early treatment with MPH may exert its protective role on psychiatric comorbidity by improving emotional regulation.

## 2. Materials and Methods

According to a naturalistic approach, 100 adult patients (age ≥ 18 years) diagnosed with ADHD were consecutively enrolled between July 2020 and July 2022 at the outpatient service of the Psychiatry Unit 2 at Pisa University Hospital. Subjects were required to have a current diagnosis of ADHD according to the Diagnostic and Statistical Manual of Mental Disorders, 5th edition (DSM-5) [40]. The Italian version of the Diagnostic Interview for ADHD in adults (DIVA 2.0), a semi-structured, clinician-administered interview, was used to assess ADHD symptoms in childhood and adulthood, according to patient and informant reports [41]. Although the interview was based on DSM-IV criteria, DSM-5 criteria were applied for the diagnosis (i.e., ≥5/9 current criteria for inattention and/or hyperactivity/impulsivity required, and symptoms being present before the age of 12 years) [40]. Accordingly, childhood onset of symptoms and adulthood persistence were required for inclusion. Operationally, since the number of symptoms required before the age of 12 is not specified in DSM-5, a conventional threshold of at least three criteria for inattention or hyperactivity/impulsivity was considered indicative of childhood-onset, as also suggested by the later version of the DIVA. A history of psychosis and current (hypo)manic or major depressive episodes, according to DSM-5 criteria, were considered exclusionary conditions. A history of previous mood episodes was allowed. All subjects provided written informed consent for study participation. The study was carried out in accordance with the Declaration of Helsinki. The protocol was approved by the Ethical Committee of the University of Pisa on 15 March 2018 (N.12712_PERUGI).

Patients were evaluated by psychiatric trainees with at least two years of experience in neurodevelopmental disorders under senior psychiatrist supervision. Educational history, marital status, family history of mood, anxiety, neurodevelopmental disorders and SUD, history of disruptive behaviors during childhood or adolescence, current ADHD presentation and lifetime psychiatric comorbidity were investigated during the first consultation. Given the risk of missing retrospective diagnoses of disruptive behavior disorders in patients who have never been referred to specialty care before adulthood, the history of disruptive behaviors was investigated by interviewing both patients and caregivers. Disruptive behaviors included argumentative/defiant behaviors and vindictiveness as described by ODD criteria or any behavior enlisted among the criteria of CD according to DSM-5. Other psychiatric comorbidities were diagnosed according to DSM-5 criteria, except for cyclothymic disorder, where only DSM-5 criteria A-B and D-G were required. Exclusion criterion C for cyclothymic disorder was not applied, and a lifetime history of major depressive or hypomanic episodes did not rule out the diagnosis. This approach permitted the inclusion of patients with a cyclothymic background, otherwise diagnosed with major depressive disorder or bipolar disorder type 2, which experience frequent mood shifts, mild to severe, moderate symptoms and significant distress and functional impairment. In addition, an early undetermined onset of cyclothymia (<21 years old) was considered mandatory, in accordance with Akiskal and Mallya criteria for cyclothymia [42], to exclude patients with iatrogenic or residual mood swings.

Conners’ Adult ADHD Rating Scales–Observer: Screening Version (CAARS-O:SV) were used to assess current ADHD symptoms severity based on caregiver reports [43]. ADHD severity was also assessed based on patient reports using the Adult ADHD Self-Report Scale (ASRS-v1.1) [44]. Three composite scores from the Behavior Rating Inventory of Executive Function—Adult version (BRIEF-A) in its caregiver-report form were used to measure executive dysfunctions, namely the Global Executive Composite (GEC) score assessing the global deficits, the Behavioral Regulation Index (BRI) that evaluates the ability to control behavior and emotional responses, and the Metacognition Index (MI) that examine problems with working memory, monitoring, planning and organization of tasks and activities [45]. Affective temperamental traits were measured by means of the Temperament Evaluation of Memphis, Pisa, Paris, and San Diego, Münster version (TEMPS-M), a self-evaluation form including five subscales, one for each affective temperamental disposition, namely depressive, cyclothymic, hyperthymic, irritable, and anxious [46,47]. Emotional dysregulation was measured using the Reactivity, Intensity, Polarity, and Stability questionnaire in its 40-item version (RIPoSt-40), including four subscales scores, assessing affective instability, positive and negative emotionality, and emotional impulsivity, and a second-order negative emotional dysregulation score made up of affective instability, negative emotionality, and emotional impulsivity subscales [48].

All the statistical analyses were performed using R Statistical Software (Foundation for Statistical Computing, Vienna, Austria). Descriptive statistics were used to summarize the demographic and clinical characteristics of the sample. Comparative analyses were first conducted between patients: (a) first referred to specialty care (i.e., child psychiatrists or psychologists) before vs. after 18 years of age; (b) first diagnosed with ADHD before vs. after 18 years of age; (c) first treated continuously with MPH (≥12 months) before 18 years of age vs. previously untreated patients. Comparative analyses were then conducted between patients subdivided into three groups according to referral to specialty care and early MPH treatment: (1) patients first referred before 18 years of age but never treated continuously with MPH; (2) patients referred and treated continuously with MPH before 18 years of age; (3) patients never referred nor treated before adulthood. Among psychiatric comorbidities, only those showing a prevalence of at least 15% in the whole sample were selected for comparison. Student’s *t*-test (or Wilcoxon rank-sum test) was used for two-group comparisons of continuous variables, while the Analysis of Variance (ANOVA; or Kruskal Wallis test), with post-hoc Dunn test, was used for the three-group comparison. Comparative analyses of categorical variables were conducted using Pearson’s chi-squared tests (or Fisher’s exact test) with pairwise Fisher’s exact test as post-hoc analysis. False discovery rate correction (FDR) for multiple comparisons was applied to post-hoc contrasts. Shapiro-Wilk test was used for normality check. Finally, a mediation analysis was conducted by means of an R mediation package using early MPH treatment as the independent variable, emotional dysregulation as the mediator and mood disorders comorbidity as the dependent variable. The significance of the mediation effect was estimated using bootstrapping procedures, i.e., the 95% confidence interval (CI) of the indirect effect was computed based on unstandardized indirect effects from 1000 bootstrapped samples. Average causal mediation effect (ACME), average direct effect (ADE) and total effect were reported. A statistical significance threshold of *p* < 0.05 was set in all the analyses.

## 3. Results

### 3.1. Sample Characteristics

Our sample comprised 100 adult patients diagnosed with ADHD, including 71 males and 29 females, aged between 18 and 55 years (median = 22.50, interquartile range [IQR] = 19–29; see Table 1 for details). Most patients were unmarried (N = 89, 89.0%), ten were married (10.0%), and one was divorced (1.0%). Years of schooling, i.e., the number of completed years of education, ranged between 8 and 25 years, with a median of 13 (IQR = 12–13.75), and two-thirds of the sample had completed secondary school (≥13 years of schooling; N = 66, 67.4%). Almost half of the subjects had repeated at least one grade (N = 46, 46.9%).

Combined ADHD was the most common clinical presentation, followed by a predominantly inattentive presentation in approximately one-third of the sample. In contrast, predominantly hyperactive/inattentive presentation was found only in three subjects. Psychiatric comorbidity was almost ubiquitous (N = 90, 90.0%), with mood disorders being the most common comorbid conditions, followed by anxiety disorders and SUD. Multiple comorbidities were also common, with more than half of subjects showing at least two conditions among mood disorders, anxiety disorders and SUD (N = 56, 56.0%). A history of disruptive behaviors was reported by 27 patients (27.0%), 11 of which had received specific pharmacological treatment with second-generation antipsychotics during childhood or adolescence.

Overall, 40 patients had never been referred to specialty care nor diagnosed or treated before 18 (40.0%). Among them, five were treated with atomoxetine at the time of assessment, while one was treated with MPH for less than a month. On the other hand, 60 patients had been referred to specialty care during childhood or adolescence, of which only 26 had been diagnosed with ADHD before age 18 (26.0% overall; 43.3% among those referred). Twenty patients had been treated with MPH for at least 12 months during childhood or adolescence (20.0% overall; 33.3% among those referred; 76.9% among those diagnosed), while two other patients had received MPH respectively for one and five months and subsequently discontinued treatment. No patients had been treated with atomoxetine during childhood or adolescence. The duration of MPH treatment during childhood/adolescence was, on average, 5.90 years among previously treated patients (standard deviation [SD] = 3.63; median = 5, IQR = 2.75–9.25, range = 1–12). Three-fourths were still on MPH treatment (N = 15, 75.0%). Conversely, four previously referred but not treated patients had initiated MPH treatment as adults at the time of assessment, and one had initiated atomoxetine. As expected, current ADHD treatment with MPH or atomoxetine was significantly more represented in early-treated patients (N = 15/20, 75.0%) compared to both early referred but not treated patients (N = 5/40, 12.5%; *p*_FDR_ < 0.001) and patients first referred as adults (N = 6/40, 15.0%; *p*_FDR_ < 0.001; χ^2^ = 31.26; *p* < 0.001). No other pharmacological treatments for ADHD (e.g., lisdexamfetamine, guanfacine) are available in Italy.

### 3.2. Early Referral to Specialty Care

Several significant differences were evidenced between patients first referred to specialty care before 18 years of age (N = 60) and those only referred in adulthood (N = 40) (Table 2). Early referred patients were significantly younger at the time of the assessment, were more frequently unmarried, had completed less years of schooling, and had less frequently a first-degree family history of neurodevelopmental disorders compared to the others. A trend toward significance was found for gender difference, with early referred patients being more frequently males. Importantly, patients first referred to specialty care before 18 years of age also showed significantly more frequently a history of disruptive behaviors in childhood or adolescence and, by definition, had been more frequently treated for disruptive disorders during childhood or adolescence than the others. No differences were observed for current ADHD presentation, lifetime comorbidity with mood disorders, anxiety disorders and SUD, and affective temperamental traits and emotional dysregulation. However, early referred patients were characterized by significantly higher severity of hyperactivity/impulsivity symptoms according to CAARS-O:SV subscale B and greater executive dysfunctions, especially in the behavioral regulation domain, according to the BRI and the GEC score from BRIEF-A.

### 3.3. Early Diagnosis of ADHD

Significant differences between patients first diagnosed with ADHD before 18 (N = 26) and those diagnosed in adulthood (N = 74) only partially overlapped with those related to early referral to specialty care (Table 3). Early-diagnosed patients were significantly younger than the others, while no significant differences were found for gender and marital status. A trend toward significance was found for years of schooling, with early-diagnosed patients having completed less years of education. Notably, a first-degree family history of SUD was significantly more frequent in early-diagnosed patients than in the others. As before, patients first diagnosed with ADHD before 18 showed significantly more frequently a history of disruptive behaviors in childhood or adolescence and had been more frequently treated for disruptive disorders during childhood or adolescence compared to those diagnosed with ADHD in adulthood. Current ADHD presentation also significantly differed between the groups, with early diagnosed patients showing more frequently combined ADHD and those diagnosed in adulthood being more frequently affected by predominantly inattentive ADHD based on uncorrected post-hoc pairwise Fisher’s exact test (*p* = 0.028, *p*_corr_ = 0.083). Importantly, mood disorders were significantly less prevalent in early-diagnosed patients than others. The same finding was observed for bipolar or related disorders. When distinguishing between different mood disorders, a significant difference between groups was confirmed, although post-hoc contrasts only showed significant differences when not correcting for multiple comparisons. Differences were restricted to early-diagnosed patients being less frequently affected by bipolar disorder type 2 (*p* = 0.023, *p*_corr_ = 0.204) or by cyclothymic disorder (*p* = 0.008, *p*_corr_ = 0.084) than by any mood disorder compared to patients diagnosed in adulthood. No differences were observed for lifetime comorbidity with anxiety disorders and SUD. Finally, early-diagnosed patients reported significantly lower ADHD symptoms severity according to the ASRS-v1.1, lower depressive and cyclothymic temperamental traits according to TEMPS-M, and lower negative emotionality and negative emotion dysregulation according to RIPoSt-40. Almost significant differences in the same direction were also shown for metacognitive dysfunctions according to the MI from BRIEF-A, anxious temperament, and emotional impulsivity.

### 3.4. Early Treatment with MPH

As expected, differences between patients first treated continuously with MPH (≥12 months) before 18 (N = 20) and those untreated (N = 80) mostly overlapped and exceeded in magnitude those related to early diagnosis of ADHD (Table 4). Early-treated patients were significantly younger, had been more frequently treated for disruptive disorders during childhood or adolescence, were more frequently diagnosed with combined and less frequently with predominantly inattentive ADHD (*p* = 0.015, *p*_corr_ = 0.044), were less frequently affected by mood disorders or bipolar disorders, especially bipolar disorder type 2 (*p* = 0.037, *p*_corr_ = 0.329) and cyclothymic disorder (*p* = 0.004, *p*_corr_ = 0.034), and showed significantly lower ADHD symptoms severity according to both the CAARS-O:SV subscale A measuring inattentive symptoms, the CAARS-O:SV subscale D measuring non-DSM-based accessory symptoms, and the ASRS-v1.1, had significantly lower metacognitive dysfunctions according to the MI from BRIEF-A, lower depressive and cyclothymic temperamental traits according to TEMPS-M, and lower negative emotionality and negative emotion dysregulation according to RIPoSt-40. Differences in the family history of SUD, history of disruptive behaviors, global executive impairments according to GEC score, anxious temperament and affective instability only approached significance.

### 3.5. Early Referral to Specialty Care vs. Early Treatment with MPH

Several differences were evidenced between patients never referred nor treated before adulthood (N = 40), patients first referred before 18 but never treated continuously with MPH (N = 40), and patients referred and treated continuously with MPH before 18 (N = 20) (Table 5). Age differed significantly between groups, with patients treated before 18 being younger than other early referred patients (*p* = 0.030, *p*_corr_ = 0.030). Patients not referred before 18 (*p* < 0.001, *p*_corr_ < 0.001), and early referred patients also being younger than those not referred before 18 (*p* < 0.001, *p*_corr_ < 0.001). Early referred patients were also more frequently unmarried (*p* = 0.014, *p*_corr_ = 0.043) and had completed less years of schooling than patients first referred in adulthood (*p* = 0.005, *p*_corr_ = 0.015). An almost significant difference in years of schooling was also found between early treated patients and those first referred in adulthood (*p* = 0.051, *p*_corr_ = 0.077). First-degree family history of neurodevelopmental disorders also differed between groups, with patients first referred in adulthood showing a higher familiarity than those early referred (*p* = 0.023, *p*_corr_ = 0.069) and those early treated (*p* = 0.077, *p*_corr_ = 0.115). 

Patients first referred in adulthood had a significantly lower rate of disruptive behaviors during childhood or adolescence compared to both early referred (*p* < 0.001, *p*_corr_ = 0.001) and early treated patients (*p* < 0.001, *p*_corr_ = 0.001). As expected, they also had been treated significantly less frequently for disruptive disorders than early-treated patients (*p* = 0.001, *p*_corr_ = 0.002), while the difference with early referred patients did not reach significance (*p* = 0.055, *p*_corr_ = 0.082). Mood disorders, instead, were significantly less common in early treated patients compared to both early referred patients (*p* = 0.003, *p*_corr_ = 0.007) and patients not referred before 18 (*p* = 0.009, *p*_corr_ = 0.013), while no differences were found between these latter groups. Bipolar or related disorders were also significantly less common in early treated patients compared to other early referred patients (*p* = 0.003, *p*_corr_ = 0.007), while the difference with patients not referred before 18 only approached significance (*p* = 0.050, *p*_corr_ = 0.073). When distinguishing between different mood disorders, a significant difference between groups was confirmed, with post-hoc contrasts showing that early-treated patients were significantly less frequently affected by cyclothymic disorder than by any mood disorder compared to other early referred patients (*p* = 0.004, *p*_corr_ = 0.038) and, though only at the uncorrected level, to patients first referred in adulthood (*p* = 0.027, *p*_corr_ = 0.270). Similarly, when not correcting for multiple comparisons, bipolar disorder type 2 was significantly less common than any mood disorder in early treated vs. early referred patients (*p* = 0.041, *p*_corr_ = 0.370).

Most differences in symptom severity occurred between early referred but not treated patients, on the one hand, and early treated patients or patients not referred before 18. Specifically, the former showed a significantly higher severity of ADHD symptoms according to CAARS-O:SV subscales A measuring inattentive symptoms (respectively, *p* = 0.001, *p*_corr_ = 0.004; and *p* = 0.023, *p*_corr_ = 0.034), C measuring total ADHD symptoms (*p* = 0.025, *p*_corr_ = 0.037; *p* = 0.009, *p*_corr_ = 0.026), and D measuring non-DSM-based accessory symptoms (*p* < 0.001, *p*_corr_ = 0.003; *p* = 0.014, *p*_corr_ = 0.021), and greater executive dysfunctions according to the BRI (*p* = 0.021, *p*_corr_ = 0.031; *p* = 0.008, *p*_corr_ = 0.023), the MI (*p* = 0.007, *p*_corr_ = 0.020; *p* = 0.031, *p*_corr_ = 0.047) and the GEC score (*p* = 0.003, *p*_corr_ = 0.010; *p* = 0.004, *p*_corr_ = 0.007) from BRIEF-A. On the other hand, early-treated patients showed a significantly lower self-reported severity of ADHD symptoms on the ASRS-v1.1 than both early referred patients (*p* = 0.018, *p*_corr_ = 0.027) and patients not referred before 18 (*p* = 0.006, *p*_corr_ = 0.018), as well as a significantly lower negative emotionality based on RIPoSt-40 (respectively, *p* = 0.013, *p*_corr_ = 0.038; and *p* = 0.016, *p*_corr_ = 0.024). Similar differences also occurred on the RIPoSt-40 negative emotion dysregulation scale, though post-hoc contrasts were not significant (respectively, *p* = 0.017, *p*_corr_ = 0.050; and *p* = 0.076, *p*_corr_ = 0.114). In addition, cyclothymic temperamental traits measured by the TEMPS-M were significantly lower in early treated vs. early referred patients (*p* = 0.012, *p*_corr_ = 0.036).

### 3.6. Mediation Analysis

Finally, a mediation analysis was performed to assess the mediating role of negative emotionality severity measured by means of the RIPoSt-40 subscale in the relationship between treatment with MPH during childhood or adolescence (≥12 months) and comorbidity with mood disorders. The effect of early MPH treatment on likelihood of mood disorders comorbidity was significantly and fully mediated via negative emotionality, with bootstrapped unstandardized ACME (average) = −0.175 (95% CI = −0.315 to −0.050, *p* = 0.004), ADE (average) = −0.249 (95% CI = −0.510 to 0.000, *p* = 0.052), and total effect = −0.424 (95% CI = −0.671 to −0.120, *p* = 0.004). On average, the proportion of the effect of early MPH treatment on mood disorders and comorbidity mediated by negative emotionality was 0.412 (95% CI = 0.131 to 1.010).

## 4. Discussion

In our study, we examined the mutual relations between a history of childhood or adolescence referral to specialty care, diagnosis of ADHD and continuous treatment with MPH, on the one hand, and current clinical presentation, psychiatric comorbidity, and emotional dysregulation, on the other, in 100 adult outpatients with ADHD. Surprisingly, while 40 of the patients recruited had been referred for specialty care during childhood or adolescence, only 26 (43% of those referred) had received the diagnosis of ADHD, and 20 (33% of those referred) had been treated for ADHD with MPH for at least 12 months. Similar to our study, underdiagnosis and undertreatment of ADHD in Italy have been evidenced by another recent study, which estimated a median duration of untreated illness of 17 years in adults with ADHD [49]. The underdiagnosis of ADHD is well known, and several explanations have been proposed. Some individuals with low severity of symptoms, good intellectual skills, and in a supportive environment may need less clinical attention until late adolescence or early adulthood when functional requests increase. In these patients, several years may pass between the onset of symptoms and the onset of impairment [50]. On the other hand, the organization of specific services for ADHD started late in Italy compared to other European countries and proceeded in a cumbersome and incomplete manner. Only recently, the National Health Service identified regional centers for the diagnosis and treatment of ADHD. The situation is even more problematic for managing adult ADHD patients, as there are no national guidelines and centers dealing with diagnosis and treatment in adults are still very few and do not usually collaborate directly with the facilities dealing with juvenile patients [51,52]. 

In our sample, early referral to specialty care was associated with younger age at the time of our assessment, being unmarried, less schooling, greater severity of hyperactivity/impulsivity symptoms and deficits of behavioral regulation. Consistently, and in accordance with our hypothesis, a history of disruptive behaviors during childhood or adolescence was strongly associated with early referral, with the prevalence of disruptive behaviors being 8 to 9-fold higher in early referred patients compared to the others. Among early referred patients, no significant difference was found concerning disruptive behaviors between those treated or not with MPH, while treatment of disruptive disorders was to some extent more common in those also treated with MPH. In summary, externalizing features and disruptive behaviors are likely to determine childhood/adolescence referral to specialty care in patients with ADHD. 

A family history of neurodevelopmental disorders, conversely, was found to be associated with adulthood referral to specialty care. While further studies are needed to confirm and extend this result, we may hypothesize that parents with an undiagnosed neurodevelopmental disorder, especially ADHD, may recognize with more difficulty inattention and hyperactivity/impulsivity as pathological in their offspring. Conversely, parents without neurodevelopmental disorders may refer their children more frequently to specialty care when noticing ADHD symptoms. According to this hypothesis, Oliva and colleagues found that having a family history of ADHD may contribute to treatment delay [49]. Alternatively, adults whose children are diagnosed with neurodevelopmental disorders may become aware of their symptoms and be referred for the first time to adult psychiatry services only after the diagnosis is made for their children. 

Correlates of early diagnosis and treatment with MPH mostly coincided, as expected, based on the substantial overlap between the groups. Indeed, about 85% of patients diagnosed with ADHD during childhood or adolescence had been prescribed MPH, and more than three-fourths had continued medication for at least one year. However, early diagnosis of ADHD, rather than treatment, was more specifically associated with a first-degree family history of SUD. To the best of our knowledge, no previous studies highlighting this association are available so far. Nevertheless, the association between ADHD and SUD is well-known and has been long investigated [24,53,54]. It is possible that ADHD symptoms were given greater attention by clinicians in patients with relatives affected by SUD, leading to an early diagnosis in this population. 

Moreover, combined ADHD presentation was also significantly more common in early-diagnosed patients, while the predominantly inattentive presentation was more common in patients diagnosed in adulthood. This finding is consistent with previous studies showing a higher prevalence of predominantly inattentive ADHD in adult samples than in child or adolescent samples. It stresses the risk of underdiagnosis of inattentive ADHD during childhood and adolescence. Indeed, it has been previously suggested that children with less pronounced levels of externalizing behaviors and a predominantly inattentive presentation are less often clinically diagnosed [55]. Alternatively, since attention deficits are the most persistent manifestations of ADHD, even among adults who were diagnosed during childhood [56], while hyperactivity/impulsivity symptoms are more likely to remit with age [57], it could not be excluded that difference in ADHD presentation may be better explained by significantly higher age observed in patients diagnosed in adulthood. 

The most important findings of this study concern early treatment with MPH. Notably, mood disorders were found to be significantly less common in early-treated patients compared to both patients who had been referred to specialists in adulthood and patients who, despite having been referred to specialty care in childhood or adolescence, had not been prescribed treatment for ADHD. The association between lack of early treatment and mood disorders was mainly driven by cyclothymic disorder and, to some extent, bipolar disorder type 2, especially in early referred patients. Indeed, almost half of this subsample was diagnosed with cyclothymic disorder. Other recent studies from our group highlighted a large overlap and phenomenological similarity between patients diagnosed with adult ADHD and cyclothymic disorder [48,58]. Moreover, mild affective disorders, such as cyclothymia, labile personality, and minor, chronic and intermittent depressive disorders, have been diagnosed in up to 65–80% of untreated patients included in early investigations on ADHD in adults [59,60].

Given the retrospective design of our study, two different, non-exclusive explanations of the negative association between early treatment and mood disorders may be hypothesized. On the one hand, differences between treated and untreated patients among those referred to specialty care can be explained by diagnostic overshadowing: ADHD may have been masked by affective disturbances in patients, preventing MPH from being prescribed [61,62,63]. However, similar differences were observed between early-treated patients and patients first referred during adulthood. This latter finding supports the potential role of early treatment with MPH in preventing the development of mood disorders in patients with ADHD. This hypothesis is consistent with previous retrospective [33,35,36], prospective [34], and register-based investigations [37] showing reduced rates of depressive and bipolar disorders in ADHD patients who had received treatment during the developmental age.

Early treatment with MPH was also associated with lower severity of current ADHD symptoms and emotional dysregulation based on self-reported measures. Particularly, negative emotionality, defined as the propensity for experiencing more often and more easily strong negative feelings, such as sadness, worry, anxiety and dissatisfaction, was significantly lower in early-treated patients than in the other groups. Interestingly, the negative association between MPH treatment and mood disorders was fully mediated by reduced negative emotionality in early-treated patients. Even in this case, given the cross-sectional design of our study, causation cannot be inferred. However, emotional dysregulation has been previously associated with a higher persistence of ADHD symptoms in adulthood [13,14], more frequent comorbid disorders, poor social outcomes and peer rejection in children and adolescents with ADHD [64,65,66], all of which may independently contribute to the development of mood disorders.

Moreover, emotional dysregulation has been found to co-aggregate with bipolar disorder in families of children with ADHD [67], as well as to confer a greater prospective risk of developing the disorder [68]. Several studies have documented the efficacy of stimulants in the treatment of emotional symptoms in adults with ADHD, showing similar magnitude of effects on ADHD and emotional symptoms and positive associations between improvements on all domains [69,70,71,72]. While subjective emotional experiences of children and adolescents with ADHD have long been neglected, beneficial effects of stimulants have been repeatedly observed on irritability or anger-related behaviors in children with ADHD [69,73,74,75,76], even in case of comorbid disruptive behavior disorders or disruptive mood dysregulation disorder [77,78]. While further controlled studies and prospective investigations are needed, positive effects of MPH on negative emotionality in children with ADHD may be posited. They could be hypothetically related to a reduced incidence of subsequent mood disorders.

Finally, in contrast with our initial hypothesis, SUD was not overrepresented among patients being first referred to specialty care or treated during adulthood. While a protective role of MPH against the development of SUD has been supported by prospective studies [31,32], first-degree family history of SUD and a history of disruptive behaviors may have confounded our findings, given the cross-sectional retrospective design of the study. Indeed, a family history of SUD and a history of disruptive behaviors were more common in early diagnosed or treated patients. Notably, both family history of SUD [79,80,81,82] and comorbidity with disruptive behavior disorders [83,84,85,86,87] have been previously associated with the development of SUD in children and adolescents with ADHD. Accordingly, opposite effects of familial load and disruptive behaviors, on the one hand, and access to MPH treatment, on the other, could underlie the lack of differences in SUD among groups observed in our study.

As outlined so far, several limitations of this study should be acknowledged. First, the clinical setting (a tertiary psychiatry unit) may not have been representative of the whole population of patients with ADHD. Particularly, early-treated patients referred to our unit may be considered a subsample of the more heterogeneous population of patients with childhood/adolescence ADHD. Patients recruited in adult studies, indeed, usually represent a more frequently affectively ill subgroup, while, as early follow-up studies evidenced [88,89,90], a substantial group of ADHD youths will eventually develop antisocial behaviors and will be less likely to be found in adult psychiatric samples. It is thus possible that early-treated patients with more severe antisocial outcomes do not reach clinical attention in adulthood. Second, the cross-sectional study design mostly limited the assessment of psychiatric comorbidity to retrospective accounts, which may be at risk of recall bias. To limit this bias, especially in patients who had never been referred to specialty care, a history of disruptive behaviors was investigated instead of diagnosing disruptive behavior disorders.

In addition, no structured retrospective accounts of symptom severity were systematically obtained, preventing us to test hypotheses on specific childhood symptoms leading to referral, diagnosis, and treatment. In addition, as previously highlighted, no causation could be inferred from our findings on the negative association between MPH treatment and mood disorders and the mediation role of emotional dysregulation. Accordingly, our results should be interpreted cautiously, and further longitudinal prospective studies are needed. Importantly, most patients who received MPH before adulthood were still on treatment at the time of assessment, while only a minority of the others were treated at study entry. Consequently, lower severity of symptoms, including emotional dysregulation, in those with a history of early treatment could be attributed to current treatment status.

Conversely, it is unlikely that a lower prevalence of mood disorders could be attributed to current ADHD treatment. Other factors, including age, previous disruptive behaviors, or previous treatment for disruptive behaviors, may also contribute to differences in mood disorders. However, while a significant age difference was found between early referred but not treated patients and patients first referred during adulthood, the prevalence of mood disorders was equally high in both groups. Similarly, while a history of disruptive behaviors was not significantly less common in early treated and early referred but not treated patients, mood disorders were overrepresented in the latter group. The role of treatments for disruptive behaviors, instead, could not be excluded. Finally, the evaluation of emotional dysregulation facets and affective temperaments was based on self-report questionnaires, which may be biased by differences in social desirability, lack of insight or malingering attempts.

## 5. Conclusions

In our study, a low rate of early ADHD diagnosis and MPH treatment was observed in a sample of 100 adult outpatients with ADHD. Several differences between adults with or without a history of childhood/adolescence referral to specialty care, diagnosis of ADHD and continuous treatment with MPH were also evidenced. In summary, patients with a history of disruptive behaviors were more likely to have been referred to specialty care during childhood/adolescence. Moreover, early referred but untreated patients showed a higher severity of current ADHD symptoms. More importantly, early MPH treatment was associated with lower rates of mood disorders and lower severity of emotional dysregulation at the time of assessment, with negative emotionality mediating the relationship between MPH treatment and mood disorders comorbidity. Accordingly, the role of MPH treatment during the developmental age in preventing mood disorders in patients with ADHD, possibly by reducing emotional dysregulation, may be hypothesized. Our findings stress the need for increased attention to ADHD symptoms in children and adolescents in our setting. Importantly, while more than half of our patients had been referred to specialty care before adulthood, only a minority received a proper diagnosis and treatment. Patients with inattentive presentation and more internalizing symptoms, in particular, are more likely to pass undiagnosed and treated.

## Figures and Tables

**Table 1:** Characteristics of the sample (N = 100).

Variables	M ± SD/n (%)
**Sociodemographic variables**	
Age	25.44 ± 8.65
Gender (male)	71 (71%)
Marital status (unmarried)	89 (89%)
School years	13.07 ± 2.5
Number of grade repetitions	0.83 ± 1.35
**Family history (first degree)**	
Mood disorders	60 (60%)
Bipolar disorder	21 (21%)
Neurodevelopmental disorder	25 (25%)
Anxiety disorders	22 (22%)
Substance use disorders	15 (15%)
Psychotic disorders	1 (1%)
Suicide attempts	1 (1%)
**ADHD specifier**	
Combined presentation	62 (62%)
Predominantly inattentive	35 (35%)
Predominantly hyperactive/impulsive	3 (3%)
**Disruptive behaviors**	
Disruptive behaviors during childhood or adolescence	27 (27%)
Treatment for disruptive behaviors during childhood or adolescence	11 (11%)
**Mood disorders (lifetime)**	
Any mood disorder	72 (72%)
Any bipolar or related disorder	69 (69%)
Bipolar disorder type 1	3 (3%)
Bipolar disorder type 2	28 (28%)
Cyclothymic disorder	38 (38%)
Major depressive disorder	3 (3%)
**Anxiety disorders (lifetime)**	
Any anxiety disorder	41 (41%)
Panic disorder	32 (32%)
Social anxiety disorder	10 (10%)
Generalized anxiety disorder	6 (6%)
**Substance use disorders (lifetime)**	
Any substance use disorder	41 (41%)
Cannabis use disorder	30 (30%)
Alcohol use disorder	19 (19%)
Cocaine use disorder	15 (15%)
Amphetamine-type stimulant use disorder	8 (8%)
Benzodiazepine use disorder	5 (5%)
Opioid use disorder	4 (4%)
**Other comorbidity (lifetime)**	
Autism spectrum disorder	5 (5%)
Intellectual disability	4 (4%)
Tic disorders	2 (2%)
Tourette syndrome	1 (1%)
Obsessive-compulsive disorder	2 (2%)
Anorexia nervosa	3 (3%)
Bulimia nervosa	3 (3%)
Binge-eating disorder	11 (11%)
Borderline personality disorder	6 (6%)
Antisocial personality disorder	2 (2%)

Abbreviations: ADHD = attention-deficit/hyperactivity disorder; M = mean; SD = standard deviation.

**Table 2:** Differences between patients first referred to specialty care during adulthood (N = 40) and those early referred, i.e., during childhood or adolescence (N = 60). Student’s t and Wilcoxon’s r are reported as summary statistics for comparisons of continuous variables, χ^2^ for chi-squared tests. *p* < 0.05 are shown in bold.

Variables	Adulthood Referral (N = 40)	Early Referral (N = 60)	Stat	*p*
**Sociodemographic variables**	M ± SD/n (%)	M ± SD/n (%)		
Age ^§^	30.95 ± 9.84	21.77 ± 5.22	0.56	**0.000**
Gender (male)	24 (60%)	47 (78.3%)	3.08	0.079
Marital status (unmarried)	31 (77.5%)	58 (96.7%)	-	**0.006**
School years ^§^	13.72 ± 2.67	12.64 ± 2.3	0.29	**0.004**
Number of grade repetitions ^§^	0.9 ± 1.79	0.78 ± 0.96	−0.08	0.424
**Family history (first degree)**				
Mood disorders	25 (62.5%)	35 (58.3%)	0.04	0.835
Bipolar disorder	8 (20%)	13 (21.7%)	0.00	1.000
Neurodevelopmental disorder	16 (40%)	9 (15%)	6.72	**0.010**
Anxiety disorders	11 (27.5%)	11 (18.3%)	0.70	0.402
Substance use disorders	5 (12.5%)	10 (16.7%)	0.08	0.775
**Clinical features**				
ADHD specifier			-	0.438
Combined presentation	22 (55%)	40 (66.7%)		
Predominantly inattentive	17 (42.5%)	18 (30%)		
Predominantly hyperactive/impulsive	1 (2.5%)	2 (3.3%)		
Disruptive behaviors during childhood or adolescence	2 (5%)	25 (41.7%)	14.56	**0.000**
Treatment for disruptive behaviors during childhood or adolescence	0 (0%)	11 (18.3%)	-	**0.003**
**Mood disorders (lifetime)**				
Any mood disorder	31 (77.5%)	41 (68.3%)	0.60	0.440
Any bipolar or related disorder	28 (70%)	41 (68.3%)	0.00	1.000
Mood disorders			-	0.273
Bipolar disorder type 1	1 (2.5%)	2 (3.3%)		
Bipolar disorder type 2	12 (30%)	16 (26.7%)		
Cyclothymic disorder	15 (37.5%)	23 (38.3%)		
Major depressive disorder	3 (7.5%)	0 (0%)		
No mood disorders	9 (22.5%)	19 (31.7%)		
**Anxiety disorders (lifetime)**				
Any anxiety disorder	15 (37.5%)	26 (43.3%)	0.14	0.709
Panic disorder	12 (30%)	20 (33.3%)	0.02	0.896
**Substance use disorders (lifetime)**				
Any substance use disorder	16 (40%)	25 (41.7%)	0	1.000
Cannabis use disorder	9 (22.5%)	21 (35%)	1.24	0.265
Alcohol use disorder	8 (20%)	11 (18.3%)	0.00	1.000
Cocaine use disorder	7 (17.5%)	8 (13.3%)	0.08	0.775
**ADHD symptoms severity (CAARS-O:SV)**				
DSM-IV inattentive symptoms	16.22 ± 5.38	17.64 ± 5.72	−1.21	0.232
DSM-IV hyperactive/impulsive symptoms	9.81 ± 6.1	12.54 ± 5.65	−2.15	**0.035**
DSM-IV ADHD symptoms total	26.03 ± 9.93	30.18 ± 9.85	−1.96	0.053
ADHD index	21.14 ± 5.68	22.5 ± 7.48	−0.99	0.325
**Self-reported ADHD symptoms severity (ASRS)**				
Total score	47.48 ± 11.16	42.45 ± 14.46	1.72	0.089
**Executive functioning (BRIEF-A)**				
Behavioral Regulation Index (BRI)	56.97 ± 10.77	61.8 ± 12.77	−1.92	0.058
Metacognition Index (MI) ^§^	86.26 ± 13.52	88.7 ± 16.03	−0.12	0.249
Global Executive Composite (GEC) ^§^	143.24 ± 21.92	150.5 ± 26.52	−0.19	0.060
**Affective temperament (TEMPS-M)**				
Depressive temperament	23.19 ± 6.19	21.58 ± 7.23	1.03	0.305
Cyclothymic temperament ^§^	22.69 ± 6.81	22.74 ± 9.19	0.00	0.996
Hyperthymic temperament	19.19 ± 5.63	20.98 ± 6.05	−1.33	0.189
Irritable temperament	18.27 ± 6.99	19.55 ± 7.39	−0.77	0.444
Anxious temperament ^§^	20.31 ± 7.11	17.91 ± 7.1	0.13	0.202
**Emotional dysregulation (RIPoSt-40)**				
Affective instability	44.81 ± 14.91	46.14 ± 17.06	−0.36	0.721
Positive emotionality	38.36 ± 10.1	39 ± 10.91	−0.26	0.792
Negative emotionality ^§^	43.42 ± 10.49	40.23 ± 12.85	0.10	0.303
Emotional impulsivity ^§^	30.67 ± 8.59	29.75 ± 10.23	0.04	0.680
Negative emotional dysregulation	119.03 ± 30.97	116.51 ± 36.9	0.32	0.749

^§^ Wilcoxon rank-sum test used. Abbreviations: ADHD = attention-deficit/hyperactivity disorder; ASRS = Adult ADHD Self-Report Scale; BRIEF-A = Behavior Rating Inventory of Executive Function—Adult version; CAARS-O:SV = Conners’ Adult ADHD Rating Scales–Observer: Screening Version; DSM-IV = Diagnostic and Statistical Manual of Mental Disorders, IV edition; M = mean; RIPoSt-40 = Reactivity, Intensity, Polarity, and Stability questionnaire, 40-item version; SD = standard deviation; TEMPS-M = Temperament Evaluation of Memphis, Pisa, Paris, and San Diego—Münster version.

**Table 3:** Differences between patients first diagnosed with ADHD care during adulthood (N = 74) and those early diagnosed, i.e., during childhood or adolescence (N = 26). Student’s t and Wilcoxon’s r are reported as summary statistics for comparisons of continuous variables, χ^2^ for chi-squared tests. *p* < 0.05 are shown in bold.

Variables	Adulthood Diagnosis (N = 74)	Early Diagnosis (N = 26)	Stat	*p*
**Sociodemographic variables**	**M ± SD/n (%)**	**M ± SD/n (%)**		
Age ^§^	27.24 ± 8.85	20.31 ± 5.54	0.48	**0.000**
Gender (male)	49 (66.2%)	22 (84.6%)	2.33	0.127
Marital status (unmarried)	65 (87.8%)	24 (92.3%)	-	0.723
School years ^§^	13.24 ± 2.79	12.62 ± 1.39	0.17	0.083
Number of grade repetitions ^§^	0.86 ± 1.49	0.73 ± 0.83	−0.04	0.700
**Family history (first degree)**				
Mood disorders	46 (62.2%)	14 (53.8%)	0.26	0.609
Bipolar disorder	14 (18.9%)	7 (26.9%)	0.34	0.560
Neurodevelopmental disorder	20 (27%)	5 (19.2%)	0.28	0.599
Anxiety disorders	18 (24.3%)	4 (15.4%)	0.45	0.502
Substance use disorders	7 (9.5%)	8 (30.8%)	-	0.021
**Clinical features**				
ADHD specifier			-	**0.013**
Combined presentation	42 (56.8%)	20 (76.9%)		
Predominantly inattentive	31 (41.9%)	4 (15.4%)		
Predominantly hyperactive/impulsive	1 (1.4%)	2 (7.7%)		
Disruptive behaviors during childhood or adolescence	14 (18.9%)	13 (50%)	7.92	**0.005**
Treatment for disruptive behaviors during childhood or adolescence	4 (5.4%)	7 (26.9%)	-	**0.006**
**Mood disorders (lifetime)**				
Any mood disorder	60 (81.1%)	12 (46.2%)	9.97	**0.002**
Any bipolar or related disorder	57 (77%)	12 (46.2%)	7.19	**0.007**
Mood disorders			-	**0.021**
Bipolar disorder type 1	3 (4.1%)	0 (0%)		
Bipolar disorder type 2	23 (31.1%)	5 (19.2%)		
Cyclothymic disorder	31 (41.9%)	7 (26.9%)		
Major depressive disorder	3 (4.1%)	0 (0%)		
No mood disorders	14 (18.9%)	14 (18.9%)		
**Anxiety disorders (lifetime)**				
Any anxiety disorder	31 (41.9%)	10 (38.5%)	0.01	0.941
Panic disorder	25 (33.8%)	7 (26.9%)	0.16	0.689
**Substance use disorders (lifetime)**				
Any substance use disorder	30 (40.5%)	11 (42.3%)	0.00	1.000
Cannabis use disorder	20 (27%)	10 (38.5%)	0.72	0.398
Alcohol use disorder	13 (17.6%)	6 (23.1%)	-	0.567
Cocaine use disorder	13 (17.6%)	2 (7.7%)	-	0.342
**ADHD symptoms severity (CAARS-O:SV)**				
DSM-IV inattentive symptoms ^§^	17.33 ± 5.59	16.35 ± 5.71	0.10	0.312
DSM-IV hyperactive/impulsive symptoms	11.38 ± 5.98	11.74 ± 6	−0.25	0.803
DSM-IV ADHD symptoms total	28.71 ± 10.09	28.09 ± 10.08	0.26	0.799
ADHD index	22.65 ± 6.32	19.91 ± 7.97	1.50	0.144
**Self-reported ADHD symptoms severity (ASRS)**				
Total score	47.24 ± 11.92	36 ± 14.3	3.03	**0.006**
**Executive functioning (BRIEF-A)**				
Behavioral Regulation Index (BRI)	60.97 ± 11.89	57.09 ± 12.96	1.27	0.214
Metacognition Index (MI) ^§^	89.33 ± 15.35	83.26 ± 13.67	0.19	0.060
Global Executive Composite (GEC)	150.3 ± 24.74	140.35 ± 24.81	1.66	0.105
**Affective temperament (TEMPS-M)**				
Depressive temperament	23.49 ± 6.34	18.39 ± 6.96	2.77	**0.010**
Cyclothymic temperament ^§^	23.94 ± 7.51	18.78 ± 9.36	0.23	**0.021**
Hyperthymic temperament ^§^	19.79 ± 5.85	21.61 ± 6.06	−0.09	0.362
Irritable temperament ^§^	19.28 ± 7.02	18.11 ± 7.92	0.08	0.448
Anxious temperament ^§^	19.79 ± 7.09	16.11 ± 6.85	0.18	0.073
**Emotional dysregulation (RIPoSt-40)**				
Affective instability ^§^	47.37 ± 15.42	39.89 ± 17.26	0.16	0.116
Positive emotionality	39.41 ± 10.28	36.5 ± 11.22	0.98	0.336
Negative emotionality ^§^	43.69 ± 11.15	34.72 ± 12.12	0.27	**0.008**
Emotional impulsivity ^§^	31.25 ± 9.19	26.5 ± 9.92	0.18	0.081
Negative emotional dysregulation	122.79 ± 32.58	101.11 ± 35.28	2.31	**0.029**

^§^ Wilcoxon rank-sum test used. Abbreviations: ADHD = attention-deficit/hyperactivity disorder; ASRS = Adult ADHD Self-Report Scale; BRIEF-A = Behavior Rating Inventory of Executive Function—Adult version; CAARS-O:SV = Conners’ Adult ADHD Rating Scales–Observer: Screening Version; DSM-IV = Diagnostic and Statistical Manual of Mental Disorders, IV edition; M = mean; RIPoSt-40 = Reactivity, Intensity, Polarity, and Stability questionnaire, 40-item version; SD = standard deviation; TEMPS-M = Temperament Evaluation of Memphis, Pisa, Paris, and San Diego—Münster version.

**Table 4:** Differences between patients first treated during adulthood or untreated (N = 80) and those treated continuously with methylphenidate (MPH) for at least 12 months during childhood or adolescence (N = 20). Student’s t and Wilcoxon’s r are reported as summary statistics for comparisons of continuous variables, χ^2^ for chi-squared tests. *p* < 0.05 are shown in bold.

Variables	Adulthood Treatment or Untreated (N = 80)	Early Treatment with MPH (N = 20)	Stat	*p*
**Sociodemographic variables**	**M ± SD/n (%)**	**M ± SD/n (%)**		
Age ^§^	26.94 ± 9.03	19.45 ± 1.99	0.43	**0.000**
Gender (male)	54 (67.5%)	17 (85%)	1.61	0.205
Marital status (unmarried)	70 (87.5%)	19 (95%)	-	0.456
School years ^§^	13.13 ± 2.72	12.85 ± 1.35	0.09	0.383
Number of grade repetitions ^§^	0.88 ± 1.44	0.65 ± 0.88	0.03	0.740
**Family history (first degree)**				
Mood disorders	49 (61.3%)	11 (55%)	0.07	0.799
Bipolar disorder	16 (20%)	5 (25%)	-	0.759
Neurodevelopmental disorder	22 (27.5%)	3 (15%)	0.75	0.386
Anxiety disorders	18 (22.5%)	4 (20%)	-	1.000
Substance use disorders	9 (11.2%)	6 (30%)	-	0.072
**Clinical features**				
ADHD specifier			-	**0.019**
Combined presentation	45 (56.3%)	17 (85%)		
Predominantly inattentive	33 (42.3%)	2 (10%)		
Predominantly hyperactive/impulsive	2 (2.5%)	1 (5%)		
Disruptive behaviors during childhood or adolescence	18 (22.5%)	9 (45%)	3.05	0.081
Treatment for disruptive behaviors during childhood or adolescence	5 (6.2%)	6 (30%)	-	**0.008**
**Mood disorders (lifetime)**				
Any mood disorder	64 (80%)	8 (40%)	10.79	**0.001**
Any bipolar or related disorder	61 (76.2%)	8 (40%)	8.21	**0.004**
Mood disorders			-	**0.018**
Bipolar disorder type 1	3 (3.8%)	0 (0%)		
Bipolar disorder type 2	24 (30%)	4 (20%)		
Cyclothymic disorder	34 (42.5%)	4 (20%)		
Major depressive disorder	3 (3.8%)	0 (0%)		
No mood disorders	16 (20%)	12 (60%)		
**Anxiety disorders (lifetime)**				
Any anxiety disorder	33 (41.2%)	8 (40%)	0.00	1.000
Panic disorder	27 (33.8%)	5 (25%)	0.23	0.630
**Substance use disorders (lifetime)**				
Any substance use disorder	33 (41.2%)	8 (40%)	0.00	1.000
Cannabis use disorder	23 (28.7%)	7 (35%)	0.07	0.785
Alcohol use disorder	15 (18.8%)	4 (20%)	-	1.000
Cocaine use disorder	14 (17.5%)	1 (5%)	-	0.292
**ADHD symptoms severity (CAARS-O:SV)**				
DSM-IV inattentive symptoms ^§^	17.74 ± 5.69	14.58 ± 4.55	0.25	**0.012**
DSM-IV hyperactive/impulsive symptoms	11.44 ± 5.9	11.58 ± 6.3	−0.09	0.931
DSM-IV ADHD symptoms total	29.18 ± 10.06	26.16 ± 9.84	1.19	0.245
ADHD index	22.96 ± 6.29	18.16 ± 7.64	2.53	**0.018**
**Self-reported ADHD symptoms severity (ASRS)**				
Total score	47.05 ± 11.77	35.31 ± 15.07	2.89	**0.009**
**Executive functioning (BRIEF-A)**				
Behavioral Regulation Index (BRI)	60.96 ± 12.29	56.32 ± 11.52	1.54	0.135
Metacognition Index (MI) ^§^	89.08 ± 15.59	82.89 ± 12.28	0.20	**0.044**
Global Executive Composite (GEC) ^§^	150.04 ± 25.29	139.21 ± 22.51	0.19	0.056
**Affective temperament (TEMPS-M)**				
Depressive temperament	23.24 ± 6.48	18.69 ± 7.02	2.34	**0.029**
Cyclothymic temperament ^§^	23.82 ± 7.61	18.56 ± 9.32	0.23	**0.024**
Hyperthymic temperament ^§^	19.67 ± 5.8	22.31 ± 6.03	−0.14	0.170
Irritable temperament ^§^	19.16 ± 6.98	18.44 ± 8.21	0.05	0.623
Anxious temperament ^§^	19.68 ± 7.21	16.06 ± 6.41	0.17	0.084
**Emotional dysregulation (RIPoSt-40)**				
Affective instability ^§^	47.44 ± 15.28	38.69 ± 17.58	0.19	0.065
Positive emotionality	39.18 ± 10.37	37 ± 11.21	0.70	0.489
Negative emotionality ^§^	43.57 ± 11.13	34.06 ± 12.21	0.27	**0.007**
Emotional impulsivity ^§^	31.13 ± 9.08	26.38 ± 10.48	0.16	0.102
Negative emotional dysregulation	122.59 ± 32.34	99.12 ± 36	2.36	**0.027**

^§^ Wilcoxon rank-sum test used. Abbreviations: ADHD = attention-deficit/hyperactivity disorder; ASRS = Adult ADHD Self-Report Scale; BRIEF-A = Behavior Rating Inventory of Executive Function—Adult version; CAARS-O:SV = Conners’ Adult ADHD Rating Scales–Observer: Screening Version; DSM-IV = Diagnostic and Statistical Manual of Mental Disorders, IV edition; M = mean; MPH = methylphenidate; RIPoSt-40 = Reactivity, Intensity, Polarity, and Stability questionnaire, 40-item version; SD = standard deviation; TEMPS-M = Temperament Evaluation of Memphis, Pisa, Paris, and San Diego—Münster version.

**Table 5:** Differences between patients first referred during adulthood (N = 40), and those early referred, i.e., during childhood or adolescence, but not treated until adulthood (N = 40), and those treated continuously with methylphenidate (MPH) for at least 12 months during childhood or adolescence (N = 20). ANOVA F-statistic or Kruskal–Wallis’ χ^2^ are reported as summary statistics for comparisons of continuous variables, χ^2^ for chi-squared tests. *p* < 0.05 are shown in bold. Groups significantly differing at post-hoc tests are identified by different subscript letters.

Variables	Adulthood Referral, Untreated (N = 40)	Early Referral, Untreated (N = 40)	Early Treatment with MPH (N = 20)	Stat	*p*
**Sociodemographic variables**					
Age ^§^	30.95 ± 9.84 _a_	22.93 ± 5.93 _b_	19.45 ± 1.99 _c_	35.88	**0.000**
Gender (male)	24 (60%)	30 (75%)	17 (85%)	4.57	0.102
Marital status (unmarried)	31 (77.5%) _a_	39 (97.5%) _b_	19 (95%)	-	**0.014**
School years ^§^	13.72 ± 2.67 _a_	12.54 ± 2.67 _b_	12.85 ± 1.35	8.55	**0.014**
Number of grade repetitions ^§^	0.9 ± 1.79	0.85 ± 1	0.65 ± 0.88	1.17	0.556
**Family history (first degree)**					
Mood disorders	25 (62.5%)	24 (60%)	11 (55%)	0.31	0.855
Bipolar disorder	8 (20%)	8 (20%)	5 (25%)	-	0.900
Neurodevelopmental disorder	16 (40%)	6 (15%)	3 (15%)	8	**0.018**
Anxiety disorders	11 (27.5%)	7 (17.5%)	4 (20%)	-	0.605
Substance use disorders	5 (12.5%)	4 (10%)	6 (30%)	-	0.130
**Clinical features**					
ADHD specifier				-	0.052
Combined presentation	22 (55%)	23 (57.5%)	17 (85%)		
Predominantly inattentive	17 (42.5%)	16 (40%)	2 (10%)		
Predominantly hyperactive/impulsive	1 (2.5%)	1 (2.5%)	1 (5%)		
Disruptive behaviors during childhood or adolescence	2 (5%) _a_	16 (40%) _b_	9 (45%) _b_	16.54	**0.000**
Treatment for disruptive behaviors during childhood or adolescence	0 (0%) _a_	5 (12.5%)	6 (30%) _b_	-	**0.001**
**Mood disorders (lifetime)**					
Any mood disorder	31 (77.5%) _a_	33 (82.5%) _a_	8 (40%) _b_	12.95	**0.002**
Any bipolar or related disorder	28 (70%)	33 (82.5%) _a_	8 (40%) _b_	11.29	**0.004**
Mood disorders				-	**0.028**
Bipolar disorder type 1	1 (2.5%)	2 (5%)	0 (0%)		
Bipolar disorder type 2	12 (30%)	12 (30%)	4 (20%)		
Cyclothymic disorder	15 (37.5%)	19 (47.5%)	4 (20%)		
Major depressive disorder	3 (7.5%)	0 (0%)	0 (0%)		
No mood disorders	9 (22.5%)	7 (17.5%)	12 (60%)		
**Anxiety disorders (lifetime)**					
Any anxiety disorder	15 (37.5%)	18 (45%)	8 (40%)	0.48	0.788
Panic disorder	12 (30%)	15 (37.5%)	5 (25%)	1.08	0.583
**Substance use disorders (lifetime)**					
Any substance use disorder	16 (40%)	17 (42.5%)	8 (40%)	0.06	0.969
Cannabis use disorder	9 (22.5%)	14 (35%)	7 (35%)	1.79	0.409
Alcohol use disorder	8 (20%)	7 (17.5%)	4 (20%)	-	1.000
Cocaine use disorder	7 (17.5%)	7 (17.5%)	1 (5%)	-	0.415
**ADHD symptoms severity (CAARS-O:SV)**					
DSM-IV inattentive symptoms ^§^	16.22 ± 5.38 _a_	19.22 ± 5.66 _b_	14.58 ± 4.55 _a_	11.53	**0.003**
DSM-IV hyperactive/impulsive symptoms	9.81 ± 6.1	13.03 ± 5.32	11.58 ± 6.3	2.78	0.067
DSM-IV ADHD symptoms total	26.03 ± 9.93 _a_	32.24 ± 9.32 _b_	26.16 ± 9.84 _a_	4.51	**0.014**
ADHD index	21.14 ± 5.68 _a_	24.73 ± 6.42 _b_	18.16 ± 7.64 _a_	7.08	**0.001**
**Self-reported ADHD symptoms severity (ASRS)**					
Total score	47.48 ± 11.16 _a_	46.54 ± 12.63 _a_	35.31 ± 15.07 _b_	5.56	**0.006**
**Executive functioning (BRIEF-A)**					
Behavioral Regulation Index (BRI)	56.97 ± 10.77 _a_	64.62 ± 12.59 _b_	56.32 ± 11.52 _a_	4.96	**0.009**
Metacognition Index (MI) ^§^	86.26 ± 13.52 _a_	91.68 ± 17.04 _b_	82.89 ± 12.28 _a_	8.71	**0.013**
Global Executive Composite (GEC) ^§^	143.24 ± 21.92 _a_	156.3 ± 26.82 _b_	139.21 ± 22.51 _a_	12.07	**0.002**
**Affective temperament (TEMPS-M)**					
Depressive temperament	23.19 ± 6.19	23.3 ± 6.92	18.69 ± 7.02	2.96	0.058
Cyclothymic temperament ^§^	22.69 ± 6.81	25.12 ± 8.37 _a_	18.56 ± 9.32 _b_	6.30	**0.043**
Hyperthymic temperament	19.19 ± 5.63	20.21 ± 6.04	22.31 ± 6.03	1.51	0.227
Irritable temperament	18.27 ± 6.99	20.18 ± 6.96	18.44 ± 8.21	0.58	0.561
Anxious temperament ^§^	20.31 ± 7.11	18.96 ± 7.37	16.06 ± 6.41	3.34	0.188
**Emotional dysregulation (RIPoSt-40)**					
Affective instability	44.81 ± 14.91	50.56 ± 15.4	38.69 ± 17.58	2.95	0.059
Positive emotionality	38.36 ± 10.1	40.14 ± 10.78	37 ± 11.21	0.48	0.619
Negative emotionality	43.42 ± 10.49 _a_	43.75 ± 12.03 _a_	34.06 ± 12.21 _b_	4.40	**0.016**
Emotional impulsivity ^§^	30.67 ± 8.59	31.68 ± 9.76	26.38 ± 10.48	2.81	0.245
Negative emotional dysregulation	119.03 ± 30.97	126.81 ± 33.99	99.12 ± 36	3.55	**0.034**

^§^ Kruskal Wallis test used. Abbreviations: ADHD = attention-deficit/hyperactivity disorder; ASRS = Adult ADHD Self-Report Scale; BRIEF-A = Behavior Rating Inventory of Executive Function—Adult version; CAARS-O:SV = Conners’ Adult ADHD Rating Scales–Observer: Screening Version; DSM-IV = Diagnostic and Statistical Manual of Mental Disorders, IV edition; M = mean; MPH = methylphenidate; RIPoSt-40 = Reactivity, Intensity, Polarity, and Stability questionnaire, 40-item version; SD = standard deviation; TEMPS-M = Temperament Evaluation of Memphis, Pisa, Paris, and San Diego—Münster version.

## Data Availability

Data is available on reasonable request from the corresponding author.
